# Graphene Phase Modulators Operating in the Transparency Regime

**DOI:** 10.1021/acsnano.4c02292

**Published:** 2024-10-22

**Authors:** Hannah F. Y. Watson, Alfonso Ruocco, Matteo Tiberi, Jakob E. Muench, Osman Balci, Sachin M. Shinde, Sandro Mignuzzi, Marianna Pantouvaki, Dries Van Thourhout, Roman Sordan, Andrea Tomadin, Vito Sorianello, Marco Romagnoli, Andrea C. Ferrari

**Affiliations:** † Cambridge Graphene Centre, University of Cambridge, 9 JJ Thomson Avenue, Cambridge CB3 0FA, U.K.; ‡ IMEC, Kapeldreef 75, Leuven B-3001, Belgium; § Politecnico di Milano, Polo di Como, Via Anzani 42, Como 22100, Italy; ∥ Dipartimento di Fisica, Università di Pisa, Largo Bruno Pontecorvo 3, Pisa 56127, Italy; ⊥ Photonic Networks and Technologies Lab, CNIT, Pisa 56124, Italy

**Keywords:** graphene, photonics, modulators, optoelectronics, layered materials.

## Abstract

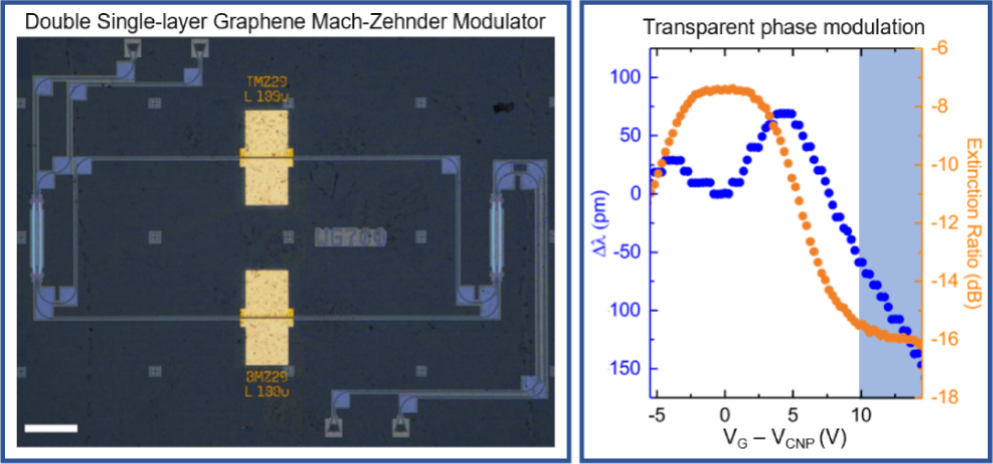

Next-generation data networks need to support Tb/s rates. In-phase and quadrature (IQ) modulation combine phase and intensity information to increase the density of encoded data, reduce overall power consumption by minimizing the number of channels, and increase noise tolerance. To reduce errors when decoding the received signal, intersymbol interference must be minimized. This is achieved with pure phase modulation, where the phase of the optical signal is controlled without changing its intensity. Phase modulators are characterized by the voltage required to achieve a π phase shift, *V*_π_, the device length, *L*, and their product, *V*_π_*L*. To reduce power consumption, IQ modulators are needed with <1 V drive voltages and compact (sub-cm) dimensions, which translate in *V*_π_*L* < 1Vcm. Si and LiNbO_3_ (LN) IQ modulators do not currently meet these requirements because *V*_π_*L* > 1Vcm. Here, we report a double single-layer graphene (SLG) Mach–Zehnder modulator (MZM) with pure phase modulation in the transparency regime, where optical losses are minimized and remain constant with increasing voltage. Our device has *V*_π_*L* ∼ 0.3Vcm, matching state-of-the-art SLG-based MZMs and plasmonic LN MZMs, but with pure phase modulation and low insertion loss (∼5 dB), essential for IQ modulation. Our *V*_π_*L* is ∼5 times lower than the lowest thin-film LN MZMs and ∼3 times lower than the lowest Si MZMs. This enables devices with complementary metal-oxide semiconductor compatible V_π_*L* (<1Vcm) and smaller footprint than LN or Si MZMs, improving circuit density and reducing power consumption by 1 order of magnitude.

The global Internet traffic was expected to triple between 2019 and 2024 with the advent of 5G and the Internet of everything.^1^ Lockdowns in response to COVID-19 shifted the distribution of data traffic across the network,^2^ with an additional ∼20–200% rise,^3^ due to remote working^3,4^ and increased use of home entertainment services.^5^ This vast amount of data relies on a backbone of high-density data network infrastructures, with 2018 standards of 400Gb/s^6^ to be extended >1Tb/s by 2025.^7^ To go >1Tb/s it is preferable to increase data rates in a single channel^8,9^ rather than the number of channels. By minimizing the number of channels, the power consumption and system complexity is reduced, because less electrical drivers and active optical components are needed.^8,9^ The bandwidth (BW) of a single channel that uses binary modulation formats is limited by that of the electrical interfaces used to drive the active optical components.^8,9^ These struggle to exceed 2023 standards^7^ because losses increase with increased frequencies.^10^ Consequently, for data rates >100Gb/s, binary modulation formats^11^ have been replaced by 4-level pulse-amplitude modulation (PAM).^6^ PAM uses 4 amplitude levels of the transmitted optical signal, to represent 4 symbols that correspond to 2 bits of information.^8^ Other multilevel modulation schemes, such as quadrature amplitude modulation (QAM),^11^ encode information in both phase and amplitude.^11^ Transmission systems that use only amplitude modulation (AM) are known as direct detection systems.^11^ Those that use both phase modulation (PM) and AM are known as coherent, because the phase difference between two or more signals remains constant over time.^11^ Coherent systems have a higher noise tolerance than direct detection ones, because the signal degradation from fiber dispersion can be compensated by the received signal phase.^11^

Information is transmitted by electro-optic (EO) modulators that convert an electrical signal into an optical one.^12^ This can be encoded into the intensity of the transmitted signal, known as AM, or electro-absorption modulation,^12^ and into the phase, known as PM or electro-refractive modulation.^12^ In-phase and quadrature (IQ) modulators are interferometric devices that use pure PM, with no change of amplitude, to generate the different QAM symbols.^11^ No direct AM is required to generate QAM symbols, because the interferometer converts a phase difference into a change in amplitude.^11^ To reduce intersymbol interference, therefore errors at the receiver,^11^ the symbol noise should be minimized, and symbols should be evenly spaced in the in-plane and quadrature axes.^11^ Thus, any unwanted AM will increase symbol noise, and any nonlinear PM will result in irregular symbol spacing.^11^

An important parameter for comparing phase modulators is the product of the voltage required to achieve a π phase shift, *V*_π_, and the device length, *L*.^13^ The additional optical loss resulting from inserting the device in the transmission line is the insertion loss IL = α*L*, where α is the absorption coefficient per unit length.^14^ In order to reduce overall power consumption, we need to minimize *V*_π_*L* and IL,^14^ because a lower *V*_π_*L* reduces the device area and capacitance, hence reducing the dynamic energy consumption E = CV^2^=C/4,^15^ i.e., the energy charged and discharged in a capacitor by an AC voltage with peak-to-peak voltage V_*pp*_. IL contributes to optical power loss and signal degradation. The PM figure of merit (FOM_PM_) is defined as the product of *V*_π_ and IL (FOM_PM_ = *V*_π_α*L*),^16^ whereby better phase modulators have a smaller FOM_PM_. The modulator BW is critical for Tb/s data transmission, in order to maximize the data rates that a single channel can support,^14^ which is ,^17^ where BW is in Hz and S/N is signal-to-noise ratio. e.g., a data rate of 100Gb/s in a single lane with S/*N* ≥ 20, which is the goal set by the 2023 Ethernet Alliance roadmap,^7^ requires BW ≥ 23 GHz. Table 1 compares the performance of our DSLG modulators with both graphene and non-graphene based technologies, already commercialized or showing promise for commercialization.

**Table 1:** Modulators Based on Si, III–V (InGaAsP), LN, and Graphene for IQ Modulators Design^a^

Ref.	Material	Type	IL [dB]	ER [dB]	V_π_L [Vcm]	L [cm]	Modulation speed	V_π_IL [VdB]
(18)	Si	MZM	5.4	3.6	1.4	0.2	55 GHz	38
(19)	Si	Ring modulator	3	9.8	0.52	0.2	50 GHz	8
(20)	Si	Thermo-optic PM	0.23	-	0.027	0.006	130 kHz	1
(21)	III–V/Si MOS	MZM depletion mode	-	11	0.24	0.05	27 GHz	-
(22)	III–V/Si MOS	MZM accumulation mode	1	12	0.047	0.047	100 MHz	1
(23)	III–V/Si MOS	MZM depletion mode	-	4.4	0.3	0.03	18 GHz	70
(24)	Thin-film LN	MZM	0.5	30	1.4	2	>45 GHz	0.4
(25)	Thin-film LN	MZM	7.6	20	6.7	0.5	106 GHz	102
(26)	Thin-film LN/Si	MZM	2.5	40	2.2	0.3	>70 GHz	18.5
(27)	Thin-film LN/Si	MZM	15	19	0.8	0.3	>40 GHz	19.5
(28)	Thin-film LN	Plasmonic MZM	19.5	2.5	0.23	0.0015	>10 GHz	2,990
(29)	DSLG	EAM	20	3	-	0.01	29 GHz	-
(30)	DSLG	Ring modulator	-	15	-	0.003	30 GHz	-
(31)	DSLG (flakes)	EAM	4	5	-	0.006	39 GHz	-
(32)	SLG/Si	MZM	10	35	0.28	0.03	5 GHz	62
This work	DSLG	MZM	5.6	25	0.3	0.0075	24 GHz	3

a IL does not include coupling losses, but only the excess loss of each device.

Table 1 shows silicon photonics (SiP),^18−20^ III–V (InGaAsP),^21−23^ LiNbO_3_ (LN),^24−28^ and graphene-based Electro-Absorption Modulators (EAMs)^29−31^ and PM.^32^ SiP offers a cost-effective solution for integrating electronic and photonic components in the same circuit by using existing complementary metal oxide semiconductor (CMOS) technology.^33^ Pure PM is difficult to achieve with Si modulators based on the plasma dispersion effect^18,19,34−37^ because, due to the Kramers–Kronig relations,^38^ any change in carrier concentration results in changes in both absorption and phase. Even if pure phase modulators in Si were to be achieved, these devices would rely on doped Si waveguides (WGs), requiring an increased optical power to overcome the additional optical losses introduced by dopants,^38^ when compared to undoped Si WGs. Other modulation mechanisms in Si can be used, such as the thermo-optic effect,^20,39^ changing the Si optical properties via electrically induced temperature changes. The thermal time constant of Si is ∼ 1 ms^40^ at room temperature (*RT*), limiting operating speeds to the kHz range.^20^ A comparison between heaters on Si photonic circuits shows that thermo-optic modulators on Si have operating speeds in the kHz range.^41^ Nonlinear effects, such as the Kerr effect,^12^ produce a change in refractive index proportional to the product of nonlinear refractive index and intensity of the propagating light.^12^ But, at telecom wavelengths (1.3, 1.5 μm), this is ∼3 orders of magnitude weaker than the plasma dispersion effect.^38^ Thus, new materials with higher nonlinear refractive index are needed.

Hybrid approaches that incorporate III–V compounds^21−23,42^ with doped Si WGs reduce *V*_π_*L* by utilizing other effects, such as band-filling,^43^ which results in reduced absorption due to occupied energy states.^44^ III–V/Si metal-oxide-semiconductor (MOS) Mach–Zehnder modulators (MZM) operating in accumulation mode,^45^ which rely on the change in accumulated charge carriers within the MOS capacitor by applying a gate voltage, have the lowest *V*_π_*L* ∼ 0.047 Vcm to date,^22^ with IL ∼ 1 dB,^22^ but are BW limited to ∼100 MHz.^22^ III–V/Si MOS MZMs struggle to maintain *V*_π_*L* = 0.047 Vcm with a higher BW, because of the high (∼3kΩ μm^22^) contact resistance^22^ to the Si electrode in the MOS configuration, with more moderate values of *V*_π_*L* = 0.24–0.3 Vcm^21,23^ for III–V MZMs operating in depletion mode with BW up to ∼27 GHz.^21^ III–V based MZMs offer a lower *V*_π_*L* compared to Si MZMs, but at the cost of more complex fabrication, with expensive III–V processing.^46^ Cost-effectiveness is determined by the cost per Watt used to manufacture III–V devices, which is 40/W to 100/W at 2018 prices^47,48^ at least 2 orders of magnitude higher than Si manufacturing.^47,48^

Integrating LiNbO_3_ (LN) on undoped Si WGs enables pure PM, exploiting the Pockel’s effect,^11^ producing a change in refractive index proportional to the electric field. Modulators based on sub-μm thin-film LN,^24−27,49^ have IL < 1 dB^24^ and BW > 100 GHz.^24,25^ Thin-film LN MZMs were reported with *V*_π_*L* ∼ 1.4 Vcm,^24^ a factor of 2 larger than state-of-the-art Si plasma-dispersion MZMs.^18^ However, this *V*_π_*L* means that cm long devices are needed to reduce *V*_π_ to CMOS compatible levels <1 V.^50^ Thin-film LN MZMs with *V*_π_*L* ∼ 0.8Vcm^27^ were demonstrated in the visible range, but with IL ∼ 15 dB.^27^ Plasmonic LN modulators show *V*_π_*L* ∼ 0.23Vcm,^28^ but with IL ∼ 19.5 dB.^28^ Modulators with lower *V*_π_*L* and IL are essential to increase the density of SiP integrated circuits, thus reducing power consumption by minimizing electrical interconnects. The interconnect losses are frequency (*f*) dependent (^51^ ) due to increased resistance caused by the skin effect,^51^ where more of the current flows at the surface as *f* increases.^52^ Therefore, for electrical interfaces driving Tb/s data rates, the power consumption of interconnects becomes the limiting factor.^10,51,53^

Graphene is ideal for optoelectronics^54−57^ due to its high carrier mobility (μ > 50000 cm^2^/V s) at *RT*,^58,59^ electrically tunable optical conductivity,^60,61^ and wavelength independent absorption in the visible (500 nm) to mid-infrared (10 μm).^61,62^ The gapless band structure with massless Dirac Fermions in single-layer graphene (SLG) enables the optical conductivity to be electrostatically controlled^60,61^ , and absorption to be suppressed.^63^ Double SLG (DSLG) phase modulators can reach a theoretical *V*_π_*L* ∼ 0.1 Vcm,^64^ which enables mm long devices with driving voltages <1 V. When absorption is suppressed, the optical losses can be reduced by orders of magnitude from >1000 dB/cm^64^ to <10 dB/cm.^64^ The combination of mm lengths and <10 dB/cm optical losses leads to IL < 1 dB, therefore minimizing power consumption. SLG can be produced at wafer scale.^56,65,66^ Chemical vapor deposition (CVD) can be used to grow polycrystalline films up to 30″^67^ or single crystals.^68^ The latter allows one to fabricate devices at predetermined locations.^29,69^ SLG films can be integrated in the CMOS back-end-of-line for wafer scale processing after fabrication of the integrated circuits.^70^ This can reduce cost and complexity of fabrication, by removing the need for doped Si WGs in DSLG designs.^29,30,71−73^ EAMs,^29,31,71,73,74^ and electro-refractive modulators^30,32,72,75^ (ERMs) based on one or more SLG have been reported, with *V*_π_*L* ∼ 0.28 *V*cm^32^ and data transmission rates ∼50Gb/s.^29^ In SLG-Si modulators, doped Si is used as one plate of the capacitor and this has two main problems: 1) Si dopants increase losses;^76^ 2) The Si mobility (∼1,400 cm^2^/Vs)^77^ is lower than SLG, hence limiting operational BW. Among ERMs, pure PM with negligible amplitude modulation was not reported so far in graphene-based devices, to the best of our knowledge.

The SLG conductivity σ(ω), derived from the Kubo formula,^78^ is a function of the angular frequency of light (ω), SLG transport relaxation time (τ), SLG Fermi level (*E*_F_), and temperature *T*:^79−81^
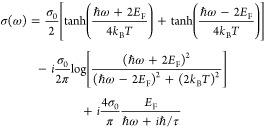
1where σ_0_=*e*/4*ℏ* is the *f*-independent, or universal conductivity of SLG,^62,82^ ℏ is the reduced Planck’s constant, and *k*_*B*_ is the Boltzmann constant. The first two terms represent interband transitions.^83,84^ The third represents intraband transitions,^83,84^ and it is a function of σ_0_, *E*_F_, ω and τ. The intraband contribution to σ(ω) can be simplified to express the DC conductivity of SLG (σ_d.c._) when ω → 0.^57^ τ can then be related to the mobility μ by using σ_d.c._=*ne*μ,^85^ where *n* is the carrier concentration given by *E*_F_ = .^79,80,86^ We thus arrive at *E*_F_(57) for *E*_F_ ≫ *k*_*B*_*T*, where  is the Fermi velocity.^79,80,83^Equation 1 implies that σ(ω) of each SLG depends on *E*_F_, and the energy of the incident light (*E*_in_ = *hc*/λ). Absorption in undoped SLG is dominated by interband transitions and is suppressed when 2*E*_F_ > *hc*/λ, due to Pauli blocking.^63^ For λ = 1.55 μm, or *E*_in_ = 0.8 eV, Pauli blocking occurs for *E*_F_ > 0.4 eV.

For Pauli blocking, SLG enters the transparency regime, whereby interband transitions are suppressed and only intraband transitions occur.^63^ Intraband transitions dominate for low energy photons (ω < 2000 cm^–1^^61^ ) and for 2*E*_F_>*hc*/λ. Intraband transitions are dependent on τ because they depend on scattering centers (e.g., defects) for conservation of momentum.^87^ Therefore, absorption by intraband transitions increases for shorter τ, which is related to mobility .^57^

Operating beyond Pauli blocking is essential for pure PM, because in this regime SLG absorption is minimized and constant with respect to gate voltage, thus reducing the overall IL. A DSLG modulator can work as EAM or ERM depending on bias.^88^ For EAMs, the onset of Pauli blocking results in the largest change in absorption,^74^ hence the bias should be set at the onset of Pauli blocking. For ERMs, the bias is set beyond the Pauli blocking condition, where the change in refractive index is quasi-linear^75^ and absorption is minimized.^64^

Here, we report DSLG-based MZMs on undoped Si WGs operating beyond Pauli blocking with *V*_π_*L* ∼ 0.3 Vcm and pure PM. These work at 16 V without dielectric breakdown, enabling access to the transparency regime. This work represents a key step in the development of graphene-based coherent integrated transmitters for communication systems.

## Results and Discussion

**Figure 1:**
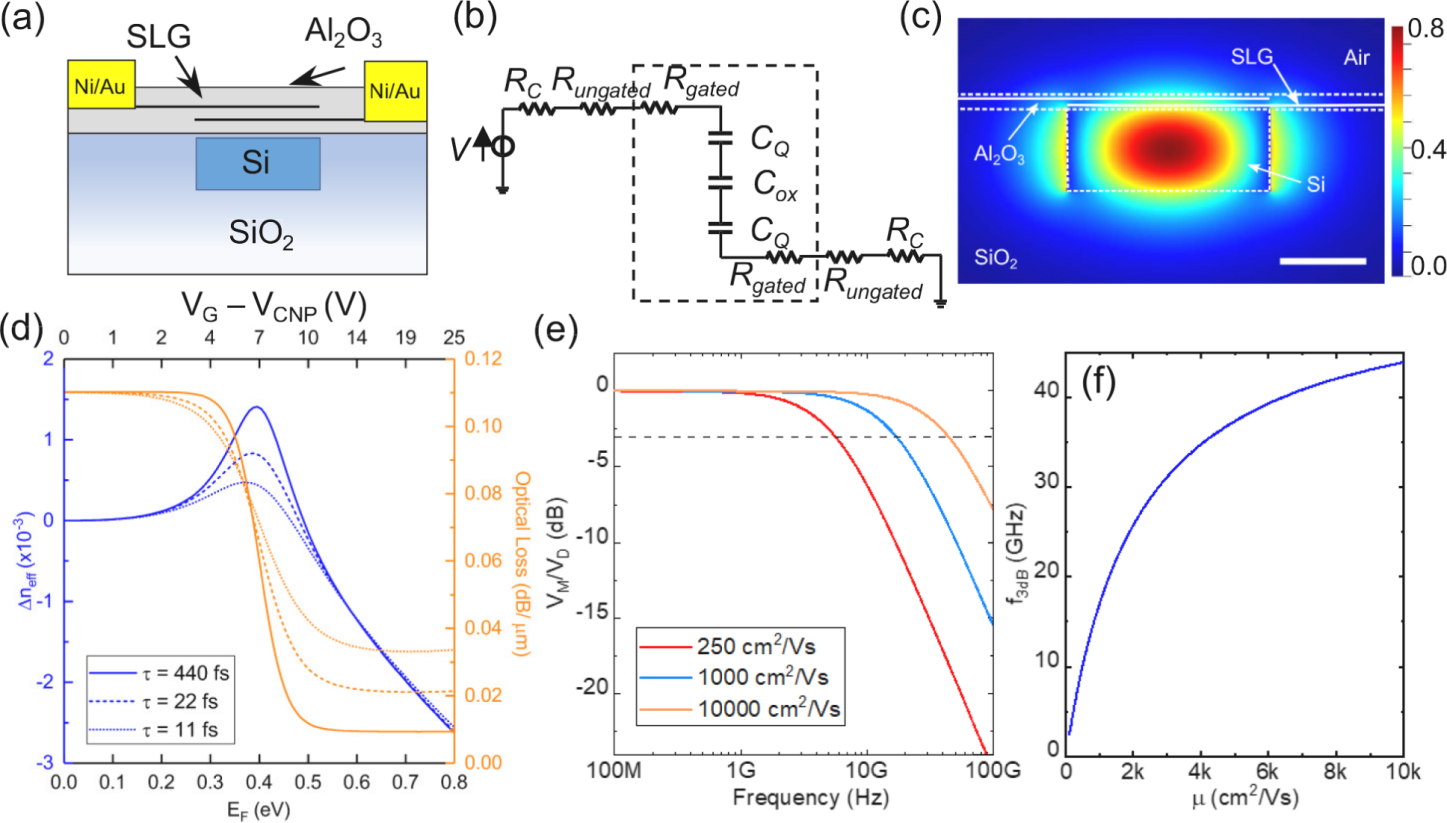
(a) DSLG modulator scheme, with 20 nm Al_2_O_3_ between bottom and top SLG, and fully encapsulating the modulator. (b) Equivalent circuit, used to calculate *Z*_T_(ω), *Z*_C_(ω), and 3 dB cutoff BW *f*_3dB_. The components contained within the dashed line contribute to the impedance of the overlapping SLG section. (c) Simulated *E*_*x*_ of fundamental TE mode confined within a 550 × 220 nm^2^ Si WG at 1.55 μm. Color scale indicates the *E*_*x*_ amplitude, scale bar 200 nm. (d) Simulated change in *n*_eff_ (blue) and optical loss (orange) of confined mode due to different *V*–*V*_CNP_ across the DSLG capacitor. Simulations are performed at 1.55 μm (∼0.8 eV). SLGs are separated by 20 nm Al_2_O_3_. The overlapping SLG region is 550 nm, the ungated SLG region is 1 μm (*E*_F_ = 0.2 eV). (e) Simulated frequency response as a function of μ for a 50 μm DSLG modulator with ungated sections of each SLG ∼ 1 μm (*E*_F_ ∼ 0.23 eV), gated sections of each SLG ∼ 450 nm (*E*_F_ ∼ 0.4 eV). *R*_*C*_ ∼ 1000 Ω · μm, 20 nm Al_2_O_3_ with ϵ_r_ = 8, *C*_eq_ calculated with an additional carrier concentration ∼10^10^ cm^–2^ from defects and ∼10^11^ cm^–2^ from charged impurities. μ calculated at 0.4 eV. (f) Simulated *f* response of DSLG modulator for different μ for the same modulator specification as (e).

The design of our DSLG phase modulator is in Figure 1a It consists of two SLG encapsulated by Al_2_O_3_, overlapping in the region above the Si WG. 10 nm Al_2_O_3_ encapsulates both SLGs to protect them during subsequent processing steps, minimize contamination, and preserve μ. The bottom encapsulation is used to maintain symmetry between the two SLGs, so that both are in the same environment. Each SLG is contacted by a metal placed on either side of the WG. The two SLG layers form a capacitor (equivalent electrical circuit in Figure 1b), where an applied voltage across the contacts creates a perpendicular electric field which modulates the carrier density, thus σ(ω) of each SLG. This, in-turn, modulates the complex effective refractive index, *n*_eff_,^83,84^ leading to a change in phase and absorption of light along the propagation direction.^12^ We simulate the optical performance of our DSLG modulators using the Finite-Difference Eigenmode (FDE) solver in Lumerical.^89^ This uses the expansion method to calculate the eigenmodes and eigenvalues of Maxwell’s equations in the *f* domain.^90^ Each solution, or mode, has its own electromagnetic field profile and *n*_eff_.^12^ The real component is *n*_eff_, related to the phase, ϕ, of the light along *L* by ϕ = *k*_0_*n*_*eff*_*L*,^12^ where *k*_0_ (2π/λ_0_) is the wavenumber in free-space and *L* is the length of the DSLG modulator. The imaginary component is the extinction coefficient, κ, related to α, and λ_0_ as .^12^

The simulated light propagation along the WG shows the electric field profile, Figure 1c, of the propagating mode. The mode interaction with SLG is increased by maximizing the overlap between SLG and field profile. The metal contacts for each SLG are placed 1 μm away from the WG edge to avoid optical losses due to proximity of field profile and contacts. The EO response is simulated by varying *E*_F_ between 0–0.8 eV and extracting the change in *n*_eff_ as a function of *E*_F_. We use *E*_F_ ∼ 0.2 eV for ungated SLG, to account for the impurity doping of as prepared SLG.^32,69^ Simulations are performed at 300 K for λ = 1.55 μm with τ = 440,22,11 fs, corresponding to μ ∼ 10000, 500, 250 cm^2^/(V s) at *E*_F_ ∼ 0.4 eV. We then calculate the phase shift Δϕ = *k*_0_Δ*n*_eff_*L* and optical losses  induced by SLG for a given *L*.^12^ We relate *E*_F_ to the applied voltage, *V*, by considering the sum of the voltages across the overlapping SLG regions and the surface voltage due to the accumulated charges at each SLG electrode:^50,64^
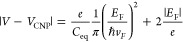
2where *V*_CNP_ is SLG charge neutrality point. The voltage across the overlapping SLG region is related to the total number of accumulated charges, *n*_tot_, and the equivalent capacitance, *C*_eq_ of the overlapping SLG region. *C*_eq_ is the series combination of the quantum capacitance,^91^*C*_*Q*_, of each SLG, and the capacitance of the parallel-plate geometry, *C*_ox_. The equivalent electrical circuit of the DSLG modulator is in Figure 1b. (92,93) and , where *d* is the thickness of the gate oxide separating the two SLG, ϵ_*r*_ is the relative permittivity of Al_2_O_3_, and ϵ_0_ is the permittivity of free space. The dielectric constant of Al_2_O_3_ is measured with a Woollam Ellipsometer *M*-2000 as . To account for charged impurities at the SLG-Al_2_O_3_ interface and the impurities introduced during growth or device fabrication, we model the total charge density *n* as the sum of a carrier concentration from electrostatic doping *n*(*V*_*G*_), with V_*G*_ the gate voltage, and that from charged impurities *n*_imp_.^92^ A charged-impurity density 10 ^12^ cm ^–2^(94) leads to an increase in carrier concentration of SLG ∼ 10 ^11^ cm^–2^.^92^ Values of impurity and carrier density are significant for identifying the ideal working point of graphene modulators, dictated by *E*_*F*_.^57^ The presence of impurities increases *n*, which changes *E*_*F*_. This cannot be neglected, because an additional carrier concentration ∼ 10^11^cm^–2^ corresponds to a change *E_F_* ∼ 0.1 eV. Hence, the charged impurity density must be considered when modeling graphene modulators to correctly identify their working point.

Figure 1d plots the simulated EO response at 1550 nm in terms of Δ*n*_eff_ and associated optical losses per μm with increasing *V*_*G*_ and *E*_F_. Optical losses decrease when *E*_F_ > 0.4 eV, corresponding to intraband transitions and the onset of Pauli blocking. For *E*_F_ > 0.4 eV, SLG enters the transparency regime, where interband transitions are blocked, such that optical losses are minimized and do not change as *E*_F_ is further increased. Δ*n*_eff_ changes sign with increasing *V*_*G*_, giving a positive or negative Δϕ for the modulated signal. A bias voltage can be applied to the DSLG modulator to define the operating point on the EO response curve in Figure 1d. The amplitude of the driving voltage defines the operating range around the operating point. The ideal working point for pure PM is in the transparency region, where Δ*n*_eff_ changes quasi-linearly, while optical losses remain constant. This also minimizes power consumption, because optical losses are at their lowest. Optical losses depend on τ, as plotted in Figure 1d for τ = 440, 22, 11 fs. A low τ is associated with high scattering rate, Γ, via ,^79^ leading to increased absorption via intraband transitions and reduced absorption via interband transitions. As *E*_F_ approaches 0.4 eV, optical losses are reduced for a lower τ, because absorption via interband transitions is reduced. However, in transparency, increased intraband transitions lead to optical losses over 3 times greater for τ = 11 fs, when compared to 440 fs.

The speed of the DSLG phase modulator is defined by the cutoff frequency, *f*_3 dB_, at which the power of the modulated signal has decreased by half (3 dB).^14^ The dominant factor that limits *f*_3 dB_ is the product of the circuit resistance, *R*, and capacitance, *C*, known as the *RC* response.^51^ We estimate this by electrical modeling, considering the different contributions to the total circuit impedance *Z*_T_(ω), coming from each contact, *R*_*C*_, ungated SLG sections, *R*_ungated_, and gated SLG sections, *R*_gated_. The equivalent circuit, Figure 1b, contains these components in series:
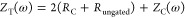
3Where *Z*_C_(ω) is the impedance of the overlapping SLG regions. *Z*_C_(ω) is given by *C*_eq_ in series with *R*_gated_ for each SLG electrode. The resistance (*R*) of SLG can be related to the sheet resistance (*R*_*S*_) of SLG as ,^54^ where *L* and *w* the length and width of SLG, respectively. By considering ω → 0, *R*_*S*_ can be related to the electrical conductivity , as .^85^*R*_*S*_ is calculated for different *E*_F_ and τ from .^54^*R*_*S*_ depends on *E*_F_, therefore on the voltage applied across the DSLG modulator. From Ohm’s law and , we calculate the frequency dependent current, *I*(ω), flowing through the circuit at a nominal drive voltage, *V*_D_, . The voltage drop across the DSLG modulator following the equivalent circuit in Figure 1b, is:
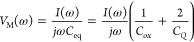
4which approaches 0 when a current *I*(ω) is flowing and 0. Therefore, by substituting *I*(ω) into , we get:
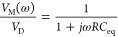
5where . Figure 1e is the frequency response of our DSLG modulators, from which the 3 dB cutoff BW can be extrapolated. *f*_3 dB_ increases with μ in Figure 1f, due to a reduction in *R*_ungated_ and *R*_gated_ for SLG with higher μ. Assuming a constant μ, *f*_3 dB_ can be increased by reducing *C* and *R*. However, there is a trade-off between minimizing ungated SLG length, to reduce *R*, and minimizing the gated SLG length, to reduce *C*. Even though *R*_ungated_ > *R*_gated_, *R*_gated_ is of the same order of magnitude as *R*_*C*_. *f*_3 dB_ can be further increased by minimizing the distance between contacts and WG, to minimize the impact from ungated regions. There is a trade-off between minimizing the required *V* to reach Pauli blocking and maximizing *f*_3 dB_. To reduce *V*, *C*_ox_ should be maximized by using a dielectric with the highest ϵ_*r*_ or reducing *d*. However, to increase *f*_3 dB_, *C*_ox_ should be reduced by increasing *d* and reducing the size of overlapping SLG region. We limit the size of overlapping SLG to the WG width and use 20 nm Al_2_O_3_ to maximize *f*_3 dB_ and limit 15 V. To operate in the transparency regime, the dielectric should support the required *V* to reach *E*_F_ > 0.4 eV without breakdown. Minimizing the size of the overlapping SLG region, we reduce the probability of breakdown due to pinholes in the dielectric.

**Figure 2:**
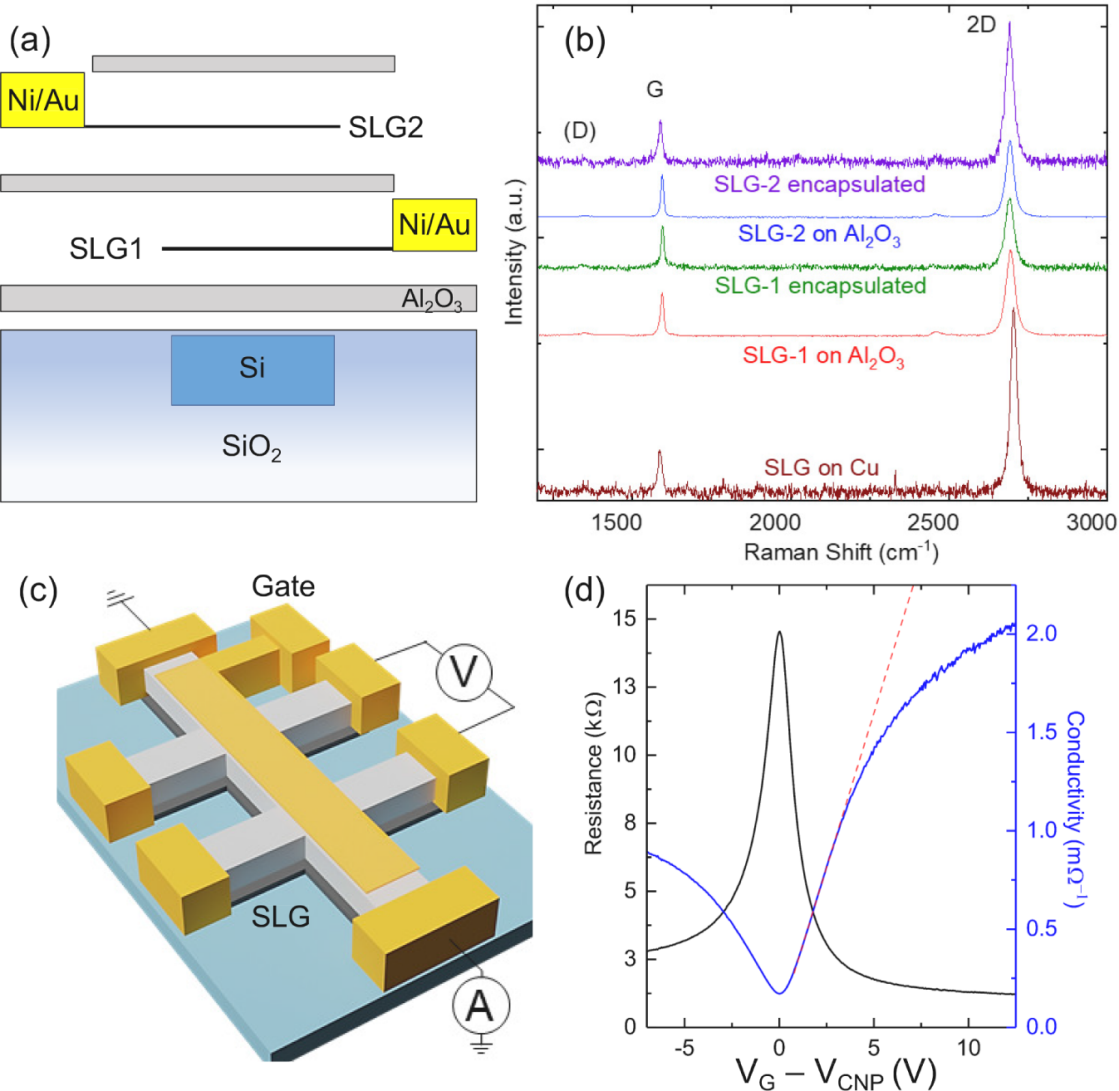
(a) DSLG modulator fabrication schematic: 10 nm Al_2_O_3_ deposition on Si WG with 20 nm Al_2_O_3_ between bottom and top SLG. 10 nm Al_2_O_3_ is used to encapsulate both SLGs to protect them during subsequent processing steps, minimize contamination, and preserve μ. The bottom encapsulation is used to maintain symmetry between the two SLGs, so that both are in the same environment. (b) Raman spectra at 514 nm for the SLG closest to the WG (SLG1) and that farthest from the WG (SLG2), as-grown on Cu, after transfer, after device fabrication. The spectra are normalized to I(G), with Cu background PL removal.^97^ (c) Schematic of SLG (gray) on SiO_2_ (green) top-gated Hall bar with Ni/Au (yellow) contacts. (d) Measured R_*S*_ (black) and calculated σ_d.c._ (σ_d.c._ = 1*R*_*S*_) (blue). Red dashed line is the σ_d.c._ linear fit for *V* > 0, showing the transition from linear to sublinear regime for *V* > 5 V.

The DSLG modulators are then fabricated as for Figure 2a. We use the IMEC silicon-on-insulator (SOI) platform because of the low (2.3 dB per grating) coupling losses.^95^ 10 nm Al_2_O_3_ is deposited on SOI by atomic layer deposition (ALD, Cambridge Nanotech Savannah S100 G1) at 120^*o*^C. After a 10 min purge of N_2_ for contaminants removal, we apply 238 consecutive cycles of 22 ms pulses of deionized water and 17 ms pulses of trimethylaluminum precursors to reach the required 10 nm thickness, as measured with a Woollam Spectroscopic Ellipsometer *M*-2000XI. Continuous SLG is grown on Cu by CVD. The Cu foil is first annealed at 1050^*o*^C under 90% H_2_ and 10% Ar at 760 Torr for 2h and cooled to *RT*. To grow SLG, the annealed Cu foil is heated to 1050^*o*^C with 40 sccm H_2_ at 0.4Torr and annealed for 2h. Growth is initiated by introducing 5 sccm CH_4_ and the CH_4_ flow is stopped to terminate growth after 30 min, and SLG/Cu is cooled to *RT*.^96^ SLG is then wet-transferred using polymethyl-methacrylate (PMMA) as a supporting layer and Cu etching in ammonium persulfate.^65^

As-grown and transferred SLG are characterized by Raman spectroscopy with a Renishaw InVia spectrometer equipped with 50× objective at 514.5 nm. Six spectra are collected from both as grown SLG on Cu and transferred SLG to estimate doping and defect density. The errors are calculated from the standard deviation across different spectra, the spectrometer resolution (∼1 cm^–1^) and the uncertainty associated with the different methods to estimate the doping from full width at half-maximum of G-peak, FWHM (G), intensity and area ratios of 2D and G peaks, I(2D)/I(G), A(2D)/A(G). Table 2 summarizes the Raman peaks fits, E_*F*_, doping type, charge carrier density *n*, strain, and defects density n_*D*_. E_*F*_ is derived from A(2D)/A(G), I(2D)/I(G) and FWHM(G). First, *n* is derived from A(2D)/A(G), I(2D)/I(G) and FWHM(G) for each spectrum by using experimental values in refs.^86,102^ producing a value for  for each spectrum. The final E_*F*_ is the average of those obtained from each spectrum. The doping type is derived from Pos(2D).^86^ For p doping, Pos(2D) increases by ∼20 cm^–1^,^86^ while for n doping there is no significant change until an electron concentration >3 × 10^13^cm^–2^ is reached.^86^ This doping dependent behavior of Pos(2D) is used to infer whether SLG is p or n doped. The strain is derived from Pos(G). Since Pos(G) depends on both E_*F*_ and strain, we first derive E_*F*_ from A(2D)/A(G), I(2D)/I(G) and FWHM(G), which are independent of strain,^63,86,102^ and then calculate Pos(G) corresponding to this E_*F*_. The strain is then retrieved from the difference between the experimental and calculated Pos(G): [Pos(G)_*calc*_-Pos(G)_*exp*_]/ΔPos(G), with ΔPos(G) ∼ 23 cm^–1^/% for uniaxial strain and ∼60 cm^–1^/% for biaxial strain.^105^ n_*D*_ is derived from I(D)/I(G) for a specific E_*F*_, using n*_D_* = (2.7 ± 0.8)  [eV]I(D)/I(G)E_*F*_ as derived in ref.^103^

**Table 2:** Raman Fit Parameters and Corresponding E_*F*_, Doping Type, *n*, Strain, n_*D*_, and Error Bars

Samples	SLG1	Encapsulated SLG1	SLG2	Encapsulated SLG2
Pos(G) (cm^–1^)	1592 ± 3	1596 ± 1	1596 ± 1	1585 ± 5
FWHM(G) (cm^–1^)	14 ± 3	12 ± 2	11 ± 2	17 ± 2
Pos(2D) (cm^–1^)	2692 ± 3	2691 ± 1	2694 ± 2	2689 ± 2
FWHM(2D) (cm^–1^)	31 ± 1	30 ± 1	29 ± 3	30 ± 4
A(2D)/A(G) (cm^–1^)	2.2 ± 0.4	5.6 ± 1.7	2.7 ± 0.1	6.3 ± 0.7
I(2D)/I(G) (cm^–1^)	2.7 ± 0.7	2.4 ± 1	1.8 ± 0.1	3.5 ± 0.5
I(D)/I(G) (cm^–1^)	0.07 ± 0.03	0.03 ± 0.04	0.05 ± 0.04	0.13 ± 0.13
E_*F*_ (meV)	190 ± 80	276 ± 158	292 ± 87	180 ± 130
Doping type	p	p	p	p
n (×10^12^) (cm^–2^)	2.6 ± 2.0	8.5 ± 9.5	5.8 ± 3.2	3.9 ± 3.8
Uniaxial strain ()	-0.20 ± 0.32	-0.15 ± 0.18	-0.07 ± 0.17	0.08 ± 0.07
Biaxial strain ()	-0.08 ± 0.14	-0.06 ± 0.07	-0.02 ± 0.06	0.003 ± 0.02
n_*D*_ (×10^10^) (cm^–2^)	2.6 ± 0.4	1.6 ± 0.9	2.5 ± 0.6	4.2 ± 2.7

The Raman spectrum of as grown SLG is in Figure 2b, after Cu photoluminescence removal.^97^ The 2D peak is a single-Lorentzian with FWHM(2D) = 27 ± 2 cm^–1^, signature of SLG.^98,99^ Pos(G) = 1591 ± 4 cm^–1^ with FWHM(G) = 16 ± 2 cm^–1^. Pos(2D) = 2712 ± 9 cm^–1^, I(2D)/I(G) ∼4.5 ± 0.7 and A(2D)/A(G)7.6 ± 1.1. No D peak is observed, indicating negligible Raman active defects.^100,101^ First, SLG1 is transferred on 10 nm Al_2_O_3_ deposited on SOI. The representative Raman spectrum of transferred SLG1 before Al_2_O_3_ encapsulation is in Figure 2b. The 2D peak retains its single-Lorentzian line shape with FWHM(2D) = 31 ± 1 cm^–1^, Pos(G) = 1592 ± 3 cm^–1^, FWHM(G) = 14 ± 3 cm^–1^, Pos(2D) = 2692 ± 3 cm^–1^, I(2D)/I(G) = 2.7 ± 0.7, and A(2D)/A(G) = 2.2 ± 0.4 indicating a p-doping with E_*F*_ = 190 ± 80 meV.^86,102^ I(D)/I(G) = 0.07 ± 0.03 corresponds to a defect density n_*D*_0.4 × 10^10^^103^ for excitation energy of 2.41 eV. SLG1 is then patterned by electron beam lithography (EBL) using a Raith EBPG5200, followed by a 60s O_2_ plasma at 10W using a Vision 320 reactive ion etcher (RIE). Contacts are fabricated using a double-layer resist mask of methyl methacrylate and PMMA,^104^ followed by 15/50 nm Ni/Au deposited by sputter coating (Precision Atomics Metallifier sputter coater) and thermal evaporation (M-Braun PROvap PVD system). A 1 nm seed-layer of Al is then thermally evaporated, before 20 nm of Al_2_O_3_ is deposited by ALD at 120^*o*^C on SLG1. After Al_2_O_3_ encapsulation, the 2D peak in SLG1 retains its single-Lorentzian line shape with FWHM(2D) = 30 ± 1 cm^–1^, Pos(G) = 1596 ± 1 cm^–1^, FWHM(G) = 12 ± 2 cm^–1^, Pos(2D) = 2691 ± 1 cm^–1^, I(2D)/I(G) = 2.4 ± 1, and A(2D)/A(G) = 5.6 ± 1.7 indicating a p-doping with E_*F*_ = 276 ± 158 meV.^86,102^ I(D)/I(G) = 0.03 ± 0.04 corresponds to n_*D*_ = 1.6 ± 0.9 × 10^10^^103^ for 2.41 eV excitation. SLG2 is transferred using the same process as SLG1, and characterized by Raman spectroscopy (Figure 2b). The 2D peak retains its single-Lorentzian line shape with FWHM(2D) = 29 ± 3 cm^–1^. Pos(G) = 1596 ± 1 cm^–1^, FWHM(G) = 11 ± 2 cm^–1^, Pos(2D) = 2694 ± 2 cm^–1^, I(2D)/I(G) = 1.8 ± 0.1, and A(2D)/A(G) = 2.7 ± 0.1, indicating a p-doping with E_*F*_ = 292 ± 87 meV.^86,102^ I(D)/I(G) = 0.05 ± 0.04 corresponds to n_*D*_ = 2.5 ± 0.6 × 10^10^^103^ for 2.41 eV. SLG2 is then patterned by using O_2_ plasma after EBL and contacts are fabricated using a double-layer resist mask for EBL as SLG1, and subsequent Ni/Au (15/50 nm) deposition. Finally, 10 nm Al_2_O_3_ is deposited on SLG2 after a 1 nm Al seed-layer is thermally evaporated. After Al_2_O_3_ encapsulation, the 2D peak in SLG2 retains its single-Lorentzian line shape with FWHM(2D) = 30 ± 4 cm^–1^, Pos(G) = 1585 ± 5 cm^–1^, FWHM(G) = 17 ± 2 cm^–1^, Pos(2D) = 2689 ± 2 cm^–1^, I(2D)/I(G) = 3.5 ± 0.5, and A(2D)/A(G) = 6.3 ± 0.7 indicating a p-doping with E_*F*_ = 180 ± 130 meV.^86,102^ I(D)/I(G) = 0.13 ± 0.13 gives n_*D*_ = 4.2 ± 2.7 × 10^10^^103^ for 2.41 eV. SLG1 and SLG2 show different doping and defect density even though they are transferred from the same SLG/Cu because SLG1 is subject to more fabrication steps than SLG2. Strain is estimated from Pos(G).^105,106^ Biaxial strain can be differentiated from uniaxial by the absence of G-peak splitting with increasing strain, however at low (≤0.5%) strain the splitting cannot be resolved. For uniaxial (biaxial) strain, Pos(G) depends on both E_*F*_ and strain.^86,105^ To obtain the contribution of strain only, we first derive E_*F*_ from A(2D)/A(G), I(2D)/I(G) and FWHM(G), which are independent of strain,^63,86,102^ and then calculate Pos(G) corresponding to this E_*F*_. The strain is then retrieved from the difference between the experimental and calculated Pos (G) (Table 2).

A 4-point-probe measurement using top-gated Hall bar structures (Figure 2c) is performed to derive SLG resistance and conductivity. Figure 2d plots the measured voltage-dependent resistance and the calculated σ_d.c._ after normalizing the conductance to the channel geometry. We observe the expected^107^ peak in resistance, which corresponds to the SLG Dirac point. μ is estimated from the measured conductivity as σ_d.c._^85,107^ where the linear region of σ_d.c._ in Figure 2d corresponds to a constant μ. The charge density *n* in terms of *C*_ox_ can be written as ,^107^ hence the conductivity becomes σ_d.c._. Using measured dielectric constant and thickness of and d = 20 nm, the measured σ_d.c._ can be fitted to estimate . The linear fit to SLG conductivity as a function of V_*G*_ is in Figure 2d.

**Figure 3:**
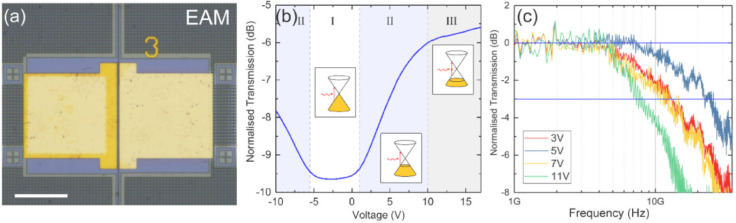
(a) Optical micrograph of EAM consisting of DSLG modulator on straight Si WGs. Scale bar 50 μm. (b) EO response of a 75 μm DSLG EAM showing the different regimes depending on *E*_F_. At 1.55 μm, transmission is lowest in region I (white), when *E*_F_ < 0.4 eV. It increases in region II (blue) due to the onset of Pauli blocking, when *E*_F_ approaches 0.4 eV, before transitioning to the minimum-loss regime in region III (gray) > 10 V, where *E*_F_ > 0.4 eV. The Si WG is 450 × 220 nm^2^. (c) EO frequency response for a 50 μm DSLG EAM at different DC biases with a 1 V peak-to-peak driving voltage. *f*_3dB_ ∼ 13 GHz for 3 V, 24 GHz for 5 V, 12 GHz for 7 V, and 8 GHz for 11 V.

The EO response of DSLG EAMs and ERMs are then measured using angled single-mode optical fibers to couple light into the photonic circuits via grating couplers. A representative EAM optical microscopy image in Figure 3a. The position of the fibers and the polarization of the source laser (Agilent 8164B Lightwave Measurement System) are adjusted to minimize coupling losses and maximize the power coupled into the confined optical mode. The steady-state response is measured by applying a DC voltage across both SLGs and measuring the optical power at the output, *P*_out_. The transmitted power, *P*_t_ = 10 log(*P*_out_/*P*_in_), is expressed in dB. Figure 3b is the optical transmission of a DSLG EAM comprising a 75 μm modulator on a straight WG. The EAM is biased between −10 and 17 V at 1.55 μm with *P*_in_ = 1 mW. To extract IL, *P*_t_ needs to be normalized to account for the additional propagation and coupling losses introduced from processing. The increase in power loss, compared to the loss before processing, is due to the deposited Al_2_O_3_ on the grating couplers, residues from SLG transfer and device fabrication. The additional losses can be subtracted by measuring the transmission through a similar WG, with same dimensions and grating couplers, that has undergone the same processing steps as the DSLG modulator. The lowest V_*G*_ dependent transmission in EAM occurs in region I in Figure 3b, between −5 and 0 V, when *E*_F_ is less than the half of the photon energy, eV. Therefore, interband transitions in SLG are allowed in region I. The transmitted power is minimum in this voltage range around *V*_CNP_ where *E*_*F*_ is closer to Dirac point.^61^ For intrinsic SLG, where *V*_CNP_ coincides with *V*_G_ = 0, the transmission curve would be centered at 0 V. In Figure 3b the *V*_CNP_ is ∼−2.5 V. This corresponds to *E*_F_ ∼ 274 meV, which represents the average E_*F*_ of both SLGs in the DSLG EAM. This is also consistent with the average SLGs *E*_F_ meV, estimated from Raman spectroscopy of SLG1 and SLG2 after Al_2_O_3_ encapsulation (Table 2, Figure 2b). As *V*_G_ increases in region II, *E*_F_ approaches , and transmission increases due to Pauli blocking of interband transitions. For *V*_G_ > 10 V, the transmission plateaus when *E*_F_ and SLG enters the transparency regime.

In transparency, IL ∼ 5.6 dB for the DSLG phase modulator, corresponding to loss ∼746 dB/cm when normalized by the modulator length. This is higher than state-of-the-art Si depletion (∼22 dB/cm),^18^ III–V (∼19 dB/cm),^22^ LN (∼0.25 dB/cm),^24^ and SLG (∼236 dB/cm^32^) MZMs (Table 1). We attribute the additional optical losses to scattering from resist residues and defects generated in SLG during fabrication, degrading τ. The simulated optical loss of the same device structure is ∼93 dB/cm for τ = 440 fs. Therefore, IL can be further reduced by improving SLG processing, increasing μ, hence reducing short-range scattering, and by developing a selective planarization process which isolates passive sections of the WGs. By modifying the thickness of the oxide layer between SLG and Si core, we can tune light-matter interactions between the Si core evanescent tail and SLG. This also applies to Cu and polymer residues which might be present. By fabricating a thick (>1 μm) oxide layer in the regions where there are no modulators (passive regions), it is possible to reduce light-matter interactions between contaminants and WG, hence reducing optical losses. At the same time, by placing a thin (<50 nm) oxide layer between SLG and WG in the regions where modulators are present (active regions), it is possible to increase ER. The size of overlapping SLG regions can be increased to further reduce IL, as the ungated SLG sections are not in transparency, hence contributing optical losses. However, any increase in the overlapping SLG region, will increase *C*_eq_, therefore will reduce *f*_3 dB_. Increasing the overlapping SLG region to ∼1 μm, would decrease optical loss to <10 dB/cm for τ = 440 fs, leading to IL < 1 dB for ∼3 mm devices, matching IL of LN^60^ and III–V^22^ MZMs (Table 1).

The EO BW, or speed, is then measured by applying a sinusoidal voltage to the DSLG modulators, in either EAM or ERM configuration. The voltage is provided by a signal generator (Agilent E8257D PSG) combined with a DC voltage via a bias tee. The optical output from the DSLG modulator is then amplified with an Er doped fiber amplifier (EDFA, Keopsys CEFA-C-HG) followed by a 1 nm narrow-band optical filter, before going into a InGaAs photodetector (PD) with a BW > 40 GHz (Newport 1014). The narrow-band filter is used to remove the noise resulting from the spontaneous emission from the EDFA,^108^ and to ensure that the PD input power is below the safe input power = 5 mW given by the specifications of the Newport PD.^109^ The modulated output signal is recorded on an electrical spectrum analyzer (ESA, Agilent PSX N9030A). By monitoring the amplitude of the modulated signal with increasing *f*, we get *f*_3 dB_. The setup is calibrated by repeating the measurements with the same configuration, but with a Thorlabs LN05S-FC AM with a 3 dB cutoff ∼40 GHz. A final normalization is then done for the *f* response of the Thorlabs LN05S-FC modulator, taken from the supplied data sheet.^110^Figure 3c is the *f* response of a 50 μm DSLG EAM for different DC biases. *f*_3 dB_ increases from 13 to 25 GHz between 3 and 5 V, then decreases to 12 GHz for 7 V, and 8 GHz for 11 V. We attribute the decrease in *f*_3 dB_ above 5 V to a reduction in μ due to increased short-range scattering of charge carriers as *E*_F_ increases. For our *RC* limited devices, we expect *f*_3 dB_ to increase with *V*, because *R*_*S*_ reduces with increasing *V*,^107^ up to an optimum point after which the increase of *C*_*q*_ becomes predominant. The optimal bias point for operating our device at 25 GHz BW is ∼5*V*, while operating it at CMOS-compatible voltages (<2 V) allows 13 GHz, Figure 3c. We assign the *V*_*G*_-dependent slow-down in Figure 3c to a decrease in μ above 5 V due to increased short-range scattering as V_*G*_ increases. This would lead to a *f*_3 dB_ slow-down of the same order of magnitude as that measured between 5 and 11 V, where *f*_3 dB_ drops from 25 to 8 GHz. This contrasts the increase from 13 to 25 GHz between 3 and 5 V, where we are still in the linear region of σ_d.c._, and benefit from decreasing *R*_*S*_. The transition to sublinear behavior can be pushed to higher V_*G*_ by decreasing the sources of short-range scattering in SLG, from SLG processing improvements. Thus, *f*_3 dB_ can be increased by improving SLG growth and transfer, to limit μ degradation during fabrication. We attribute the observed 17 GHz variation in BW at different V_*g*_ not only to the variation of μ, but also contact resistance, as we change V_*g*_. μ is voltage-dependent, with a minimum at the Dirac point (charge neutrality point V_*CNP*_). When changing the working point of the modulator by varying the driving voltage, we modify μ too.^111^ This directly impacts the RC response of the modulator, hence its BW *f*_3 dB_ = 1/2π*RC*. However, the observed variation in BW cannot be explained solely by the change in μ, as 17 GHz magnitude of variation was not previously reported for other SLG modulators, to the best of our knowledge. There is, thus, also a gate-dependent contact resistance contribution, that we observe because hundreds Ω change in resistance R could change *f*_3dB_ = 1/2π*RC* by 17 GHz.

**Figure 4:**
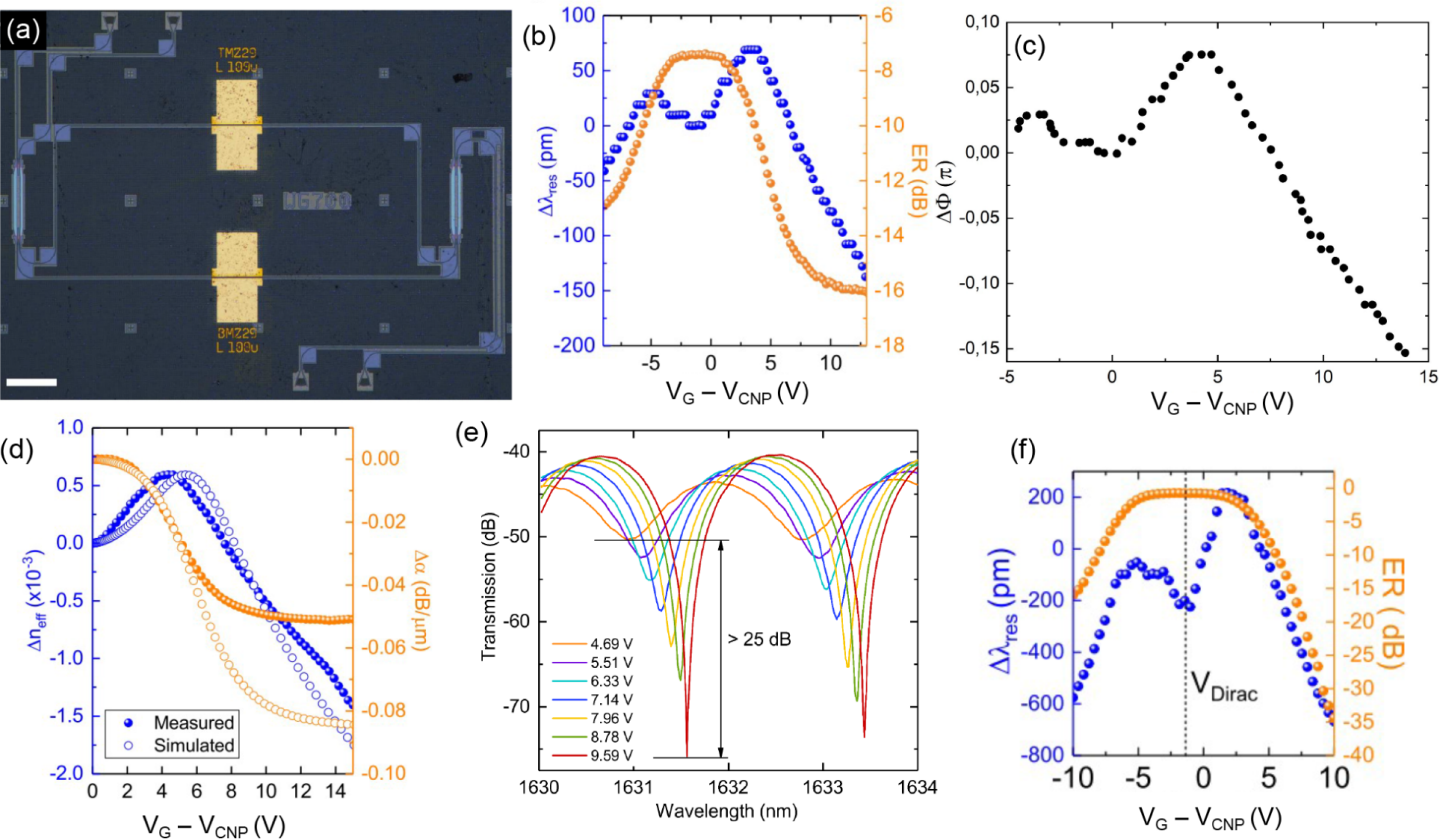
(a) Optical micrograph of MZM consisting of a DSLG modulator on each arm of a Si MZI. Scale bars 100 μm. (b) Voltage-dependent shift of interference fringe position (blue) and ER (orange) of an MZM with a ∼100 μm DSLG modulator on each arm, with one modulator biased at 10 V. The MZI is unbalanced, with 2 input and output ports, and 620 × 220 nm^2^ Si WGs. (c) Phase shift as a function of voltage for a 100 μm long modulator. (d) Comparison of measured (solid circles) and simulated (open circles) Δ*n*_eff_ (blue) and Δα (orange) for a 450 μm DSLG MZM. Simulation performed at 1.55 μm with τ = 14 fs, same device structure as the measured device, 550 × 220 nm^2^ Si WG, overlapping SLG region ∼550 nm, ungated SLG region ∼1 μm (*E*_F_ = 0.2 eV), 20 nm Al_2_O_3_ with  = 8. (e) Voltage-dependent transmission of a MZM containing one 450 μm DSLG modulator on each arm, one biased at 10 V and the other swept from 4.7 V (orange) to 9.6 V (red). (f) Voltage-dependent shift of interference fringe position (blue) and ER (orange) of an MZM with a ∼450 μm DSLG modulator on each arm, with one modulator biased at 10 V.

The simultaneous phase change that accompanies the change of amplitude cannot be extracted from an electro-absorption configuration, because the transmission of a straight WG is independent of optical signal phase.^12^ Instead, it is measured using an electro-refractive configuration, with a Mach–Zehnder interferometer (MZI).^12,112^ The optical microscopy image of a representative MZI is in Figure 4a. Here, the optical signal is split into two arms. Depending on the phase difference between these, Δϕ, the propagating waves will interfere when recombined. If the propagating waves are in phase, transmission will not depend on λ.^12^ If they are not in phase, an interference pattern will appear.^12^ In our case we have an unbalanced MZI and transmission is wavelength-dependent, hence an interference pattern will appear. This is characterized by the free spectral range ,^112^ defined as the wavelength difference between each transmission minima, where λ _res_ is the fringe position, *n*_*g*_ is the group index, and Δ*L* is the length difference between the arms of the MZI. λ _res_ depends on Δϕ, which can result from a modulator that induces Δ*n*_eff_, or when the MZI arms are different lengths, known as an unbalanced MZI.^13^ A MZM uses an ERM on one or both MZI arms to control Δϕ. Δϕ can then be directly measured by the shift of the output interference pattern.^12^ Placing an ERM on each arm enables the phase to be controlled independently on each arm to reach the required Δϕ. Figure 4b shows the V-dependent frince shift, Δλ, and extinction ratio (ER) of a MZM with a 100 μm DSLG modulator on each arm of an unbalanced MZI. One device is biased at 10 V, so that it is in the transparency regime, and the other is swept from 4 to 10 V. By measuring Δλ for different V_*G*_, we determine Δϕ, shown in Figure 4c. The measured Δλ is normalized by the FSR, which corresponds to a phase difference of 2π,^12^ giving Δϕ in units of π: .^72^ Δϕ is related to the V-induced change in the real component of *n*_eff_ along *L*, by Δϕ = *k*_0_Δ*n*_eff_*L*.^12^

The extinction ratio (ER) =,^13^ where *P*_(t,max)_ is the maximum transmitted power and *P*_(t,min)_ the minimum transmitted power, is affected by the difference in absorption between the MZI arms, Δα. Simulated and measured Δ*n*_eff_ and Δα are shown in Figure 4d. If the propagating wave in one arm is absorbed, there is no interference at the output, because there will only be one propagating wave remaining.^12^ For losses that do not result in complete absorption, ER will increase when Δα is minimized, and decrease when Δα is maximized. The MZM ER can be related to Δα by considering the transmission through the MZM as the sum of the electric fields propagating down each MZI arm: .^12,112^ ER is given by the ratio of maximum and minimum transmission through the MZM, which occurs when Δϕ = 0 and π:^12^
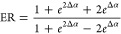
6Figure 4e,f show that, as *V*_*G*_ increases, and the SLG on the active arm becomes transparent, ER increases >25 dB because Δα is reduced. The effect of Δϕ and Δα due to the active arm of the MZM is seen by the simultaneous change in position and ER of the interference fringes with *V*_*G*_. Figure 4f plots the change in position of interference fringe and ER as a function of *V*_*G*_. The MZM has the same behavior as the EAM in Figure 3. ER is minimized near the Dirac point, because the absorption of the SLG on the active arm is highest, while the device on the other arm is transparent. ER then increases with increasing *V*_*G*_, as absorption by the active arm is reduced, until flattening >10 V, when the SLGs on both arms are transparent. This shows that the transparency regime is ideal for pure PM because we have a quasi-linear change in phase, while losses remain constant. A similar V-dependent change in fringe position and ER is observed on either side of the Dirac point, where negative V_*G*_ give a weaker effect than positive ones. The similarity around the Dirac point is due to the SLG ambipolarity,^107^ and the asymmetry can be due to different scattering rates of electrons (*e*) and holes (*h*), resulting from an uneven distribution of positively or negatively charged impurities.^94,113−115^ From Δλ and Δ*E*R we extract Δ*n*_eff_ and Δα, Figure 4d. The measured and simulated Δ*n*_eff_ and Δα are in Figure 4d. We attribute the differences in measured and simulated behavior to asymmetries between the two SLG as each SLG undergoes different amounts of processing, since SLG1 is subject to more processing than SLG2. The difference between measured and simulated Δα in transparency is a result of increased propagation losses outside the DSLG modulator, due to residues remaining on the WG from SLG processing. Values of ΔΦ calculated in transparency for a 100 μm long modulator range from −0.01π at 8 V to −0.16π at 13.8 V, which give a *V*_π_*L* ∼ 0.3 Vcm, matching that of state-of-the-art SLG PMs.^32^ However, unlike ref (32), our devices have pure PM with negligible change in optical losses, an essential property for IQ modulation.^11^*V_π_L* is obtained from ΔΦ as follows. The Mach–Zehnder interferometer free spectral range (FSR) is 1.86 nm, as derived from Figure 4e, then  using the wavelength shift in Figure 4b Δλ = 142 pm from 8 to 13.8 V. The formula used to calculate the phase shift is *L*, with Δ*n*_eff_ = λ^2^/(FSR × L). This phase shift corresponds to a modulation efficiency *V_π_L* = Δ*V*/ΔΦ *L* = 0.3 Vcm, for *L* = 100 μm, calculated in a range where SLG absorption is negligible, as shown in Figure 4b for V_*G*_-8 V. PM with negligible AM only occurs in the voltage range ∼10–13 V in Figure 4b. The modulation efficiency of 0.3Vcm also considers the phase shift which occurs in the AM regime. In addition, we directly measure up to ΔΦ = Δλ/(FSR/2) = 0.6, with Δλ = 650 pm from 5 to 10 V, in a device where AM is not negligible. The latter wavelength shift is taken from Figure 4e,f. Our modulators with *L* = 450 μm can achieve π phase shift if driven in a push–pull configuration,^116^ while increasing *L* to 0.6 cm would enable a 2π phase shift. Our DSLG MZMs have a *V*_π_*L* on par with the lowest reported plasmonic LN MZMs,^28^ ∼5 times better than the lowest reported LN MZMs,^49^ and ∼2.5 times better than the lowest reported thin film LN MZMs^27^ and Si MZMs^18^ (Table 1). Due to the high (∼746 dB/cm) optical loss, our DSLG phase modulator has FOM_PM_ > 200VdB, greater than the lowest reported Si (∼38VdB),^18^ LN (∼0.35VdB),^24^ and III–V (∼1VdB^22^) (Table 1). However, if the optical losses of SLG in transparency are reduced <10 dB/cm by increasing the overlapping SLG region and increasing τ to >300 fs, corresponding to μ > 6,000 cm^2^V^–1^s^–1^, our low *V*_π_*L* would enable FOM_PM_ ∼ 3VdB. This is a realistic perspective, because we reported an average 8,000 cm^2^/(V s) for CVD SLG transferred on Si in ref.^117^ This is 800% higher than that used here. By inserting this μ in our simulations, we get optical losses ∼9.46 dB/cm. FOM_PM_ ∼ 3VdB is lower than both Si and LN MZMs, with ∼3 mm devices instead ∼2 cm. Even though III–V MZMs have the lowest FOM_PM_ ∼ 1VdB,^22^ their BW is unsuitable for Tb/s data transmission because it is limited to the MHz range.^22^ Shrinking device dimensions by 1 order of magnitude results in denser circuits that benefit from reduced overall power consumption by minimizing the interconnects lengths.

## Conclusions

We reported DSLG MZMs showing pure PM in the transparency regime for *E*_F_ > 0.4 eV, with *V_π_L* ∼ 0.3Vcm. We reached the transparency regime by device design and process optimization, ensuring the dielectric can withstand the required 10 V to reach *E*_F_ > 0.4 eV without breakdown. Compared to SLG on Si phase modulators,^32^ our work has reduced IL from 10 to 5 dB, while maintaining the same modulation efficiency, and almost doubled BW, while operating the device in transparency (5 to 8 GHz). By operating our devices in the regime where also AM occurs, our BW is 5 times larger than ref.^32^ The decrease in optical loss is due to the DSLG structure, which does not require Si doping, thus avoiding associated losses. Indeed, the DSLG structure can be integrated on any passive photonic platform, making it an enabling low-cost technology. We measured up to π/2 phase shift, enough for applications such as binary phase shift keying.^118^ A full 2π phase shift can be achieved with *L* = 0.6 cm. Our work represents a significant step forward compared to the SLG-Si modulator architecture, since it enables the use of purely passive Si WG, hence reducing losses, with BW determined by the SLG μ.^75^ Our low *V*_π_*L* = 0.3Vcm means we are able to overcome the loss limitations of Si MZMs, deliver increased circuit densities compared to LN, and match the performance of III–V (InGaAsP) MZMs, without expensive fabrication requirements. Reaching transparency is critical for graphene-based communications and metrology platforms that use complex modulation formats to maximize the density of transmitted information.

## Methods

### Simulations

The electro-optic response is simulated with the FDE solver in Lumerical Mode, an open-source electromagnetic modeling software.^119^ SLG is modeled with the surface conductivity model available in the software (eq 1), derived from Kubo’s formalism.^78^ The frequency response is simulated by analytically solving the equivalent electrical circuit characteristics of the modulator, considering the different contributions to the total circuit impedance, , coming from both metallic contacts and SLG. For FDE simulations, we use a minimum mesh step size of 10 nm, perfect matching layer (PML) boundary conditions to minimize reflections, and a local mesh of 1 nm step, overriding the larger mesh where SLG is located.

### Fabrication

The fabrication of the DSLG modulators is as follows. First, we deposit 10 nm alumina by thermal atomic layer deposition (ALD, Cambridge Nanotech Savannah S100 G1) on the SOI platform at 120^◦^C, then we grow SLG by CVD on Cu and we transfer it on SOI using PMMA as supporting layer. SLG is shaped by oxygen plasma etching and metallized by thermal evaporation of Ni and Au after EBL (EBPG Raith 5200). Alumina deposition, SLG transfer, shaping and metallization steps are repeated to finalize the fabrication of the DSLG modulator. A final deposition of alumina is done to encapsulate the DSLG structure.

### Electrical and Optoelectronic Characterization

Electrical characterization is done by 4-probe measurements using a semiautomatic Cascade probe station on top-gated Hall Bar devices to measure resistance, conductivity, and μ. Optoelectronic characterization is done with a custom fiber-to-chip setup employing source-measure units for DC electrical probing, a telecom C-band laser, commercial photodiodes, one RF signal generator connected to 40 GHz RF probes, and an electrical spectrum analyzer to monitor the transmitted electrical power.
